# A Self-Healing Structure Based on Monolayer Wall-Less Microvascular Network Carriers for Orthotropic Anisotropic Polymer Composites

**DOI:** 10.3390/polym17060749

**Published:** 2025-03-12

**Authors:** Shenbiao Wang, Peng Li, Baijia Fan, Yuan Zhao, Shenglin Yu, Jianbin Tan, Changyou Zhang

**Affiliations:** School of Mechatronics and Vehicle Engineering, East China Jiaotong University, Nanchang 330013, China

**Keywords:** self-healing structure, orthotropic anisotropic polymer composites, light energy damage self-healing, wall-less microvascular network, non-dominated sorting genetic algorithm II (NSGA-II)

## Abstract

Due to the anisotropic structure and mechanical properties of composite laminates, internal damage cracks can easily occur. In this study, orthotropic anisotropic glass-fiber-reinforced polymer composites were used as the repair object. Firstly, the anisotropic material was analyzed using the finite element method, the self-healing structural compliance, water head loss, and volume percentage of the microvascular network were taken as the objective functions, and the topology optimization of the microvascular network structure was carried out using non-dominated soring genetic algorithm II. Secondly, the self-healing material with a wall-less microvascular network was prepared via the vacuum-assisted resin transfer molding process and the embedded wire removal method. Finally, the light repair performance was tested using the three-point bending test. The results show that in the case of no intervention for light repair, the average maximum failure load of the self-healing structure after embedding the microvascular network can reach 94.06% of that before embedding; with the introduction of real-time light repair, the average maximum failure load of the self-healing structure with light repair was increased by 4.1% compared with the self-healing structure without light repair. Meanwhile, the second peak load of the light-repaired structure can reach 51.36% of the average maximum failure load, which is 28.56% higher than that of the non-light-repaired structure.

## 1. Introduction

Glass-fiber-reinforced polymer composites have excellent properties, such as high strength, a light weight, corrosion-resistant insulation, and high fracture strain, and are widely used in many fields [1]: for example, they are widely used in the aerospace field to manufacture aircraft wings, fuselages, tail fins, etc. In the automotive industry, they are widely used in the manufacture of body shells, engine hoods, bumpers, seat skeletons, and other components. However, due to the large dispersion of the structure and the mechanical properties of glass-fiber-reinforced polymer composites, matrix cracking, fiber fracture, and delamination damage ca easily occur in the material during service [2,3], resulting in a decline in the overall performance of the material [4]. Moreover, under different strain rates, the effects on the mechanical properties of the material are significantly different [5].

In response to the above problems, inspired by bionics, researchers have proposed the concept of material damage self-healing [6], aiming to ensure the safe service of materials. A self-healing structure refers to the pre-implantation of a carrier containing a repair agent inside the material. When the material is damaged, the crack induces the rupture of the carrier and releases the repair agent, realizing the self-healing of material damage [7,8]. In various repair systems, the interconnected microvascular network carriers [9] can realize the supply and replacement of repair agents, and have the characteristics of multi-point and multi-time repair, becoming the main technical means of self-healing research [10,11]. However, in the self-healing design of anisotropic materials, it is difficult to solve the influence of embedded carriers on the mechanical properties of the structure, while ensuring the fluidity of repair agents and real-time repair.

The initial focus of self-healing research was primarily on isotropic materials, as this facilitated the design and implementation of self-healing studies. Toohey et al. [12,13] took isotropic epoxy resin materials as the research object and fabricated microvascular networks via the direct write method. He injected dicyclopentadiene as a restorative agent into the microvascular network and buried it in an epoxy-coated material containing Grubbs catalyst. Finally, he used the four-point bending test to test its restoration performance. Postiglione et al. [14] took an epoxy matrix as the research object and poured the resin into a 3D-printed water-soluble PVA mold. Finally, he prepared samples with a microvascular network and conducted damage tests on the samples. White et al. [15] investigated the effect of temperature on the repair rate based on a material with dual interpenetrating microvascular networks and a self-healing epoxy matrix. With the development of damage self-healing research, its application has gradually expanded to anisotropic materials. Nguyen [16] used progressive damage finite element analysis to study the effect of microvascular channel diameter on the structural properties of laminated plates. Li Peng et al. [17] took a glass-fiber-reinforced resin matrix composite as the self-healing object. The diameter and number of microvasculature channels were optimized based on the improved non-dominated sorting genetic algorithm, which took the tube density, the percentage of microvascular volume, and the water head loss of the repair agent as the objective functions. The fatigue test was performed to test the related properties. Williams et al. [18] studied the effect of the distribution distance of embedded hollow glass fibers (HGFs) on the repair efficiency of composite laminates. The results showed that the specimens exhibited the highest repair efficiency at a hollow glass fiber spacing of 70 μm, and the bending strength could be recovered to 97% of that of the intact specimens. However, this arrangement also causes obvious damage to the original structure of the specimen and significantly reduces other mechanical properties. Although microvascular network carriers show good self-healing properties after material damage, their structure is complex and the incorporation of microvascular networks will inevitably result in a substantial reduction in structural strength. Therefore, it is particularly important to consider the influence of microvascular network carriers on the macroscopic mechanical properties of anisotropic composites after implantation.

In addition, the current research on the carriers of self-healing materials mainly focuses on the wall microvascular network. Pang and Bond [19] used borosilicate to make hollow fibers with an outer diameter of 60 μm, which were buried into the composite, to study the repair efficiency of the material for impact damage under 24 h of repair at room temperature and 1.5 h of repair at 40 °C. Wu et al. [20] used liquid dicyclopentadiene (DCPD) as the healing agent and polyacrylonitrile (PAN) as the shell material to prepare self-healing fibers with an inner diameter of about 0.71 μm by means of coelectrospinning. The self-healing effect of core–shell nanofibers on the flexural stiffness of composite laminates after pre-damage was evaluated by means of the three-point bending test. Min et al. [21] used polyvinylidene difluoride (PVDF) as the tube wall material, epoxy resin and curing agent as the core materials, and wrapped them. After the material was damaged, the epoxy resin and curing agent leaked to heal the damaged site. Hansen et al. [22] buried two sets of pipe network carriers containing catalysts and repair agents intertwined with each other into the material to prepare damage self-healing materials, and the results of their study showed that the damaged material can recover 50% of its mechanical properties after 48 h of repair at 30 °C. Vidinejevs et al. [23] used pultruded carbon fiber-reinforced plastic micro-tubes for the self-healing of a laminated polymer composite. The interlaminar shear strength was determined via the tensile test, with epoxy resin and curing agent as repair agents. The results show that the composite material is damaged by quasi-static indentation, and after healing at 30 °C for 24 h, the interlayer shear strength of the sample can be restored to 70 ± 15% of that of the original sample.

At present, the research on damage self-healing mainly focuses on isotropic materials, and there are few studies on self-healing structure optimization considering anisotropy. In addition, in the current design of self-healing structures, wall carriers and two-component restorations are mainly used. The wall material of the carrier not only affects the mechanical compatibility of the carrier and the material but also reduces the effective volume of the microvascular network. At the same time, two-component restorations also have some disadvantages such as harsh polymerization conditions and a long repair time. The proposed self-healing structure based on monolayer wall-less microvascular network carriers for orthotropic anisotropic composites is presented in this study. A glass-fiber-reinforced polymer composite with an orthogonal anisotropic material was used as the restoration object, and the transverse and longitudinal spacing and radius of the microvascular structure were optimized by using NSGA-II with the objective functions of self-healing structural compliance, water head loss, and the volume percentage of the microvascular network. The wall-less microvascular network was prepared via the embedding line removal method. The mechanical compatibility of the microvascular network carriers and material was ensured. Simultaneously, a short-wave light-curing binder was employed as a light repair agent to induce self-healing by means of light energy [24,25]. It has the advantages of low energy consumption and high efficiency and can realize the rapid repair of composite materials in real time.

## 2. Self-Healing Topology Optimization

### 2.1. Self-Healing Structural Models for Orthotropic Anisotropic Composites

**Figure 1:**
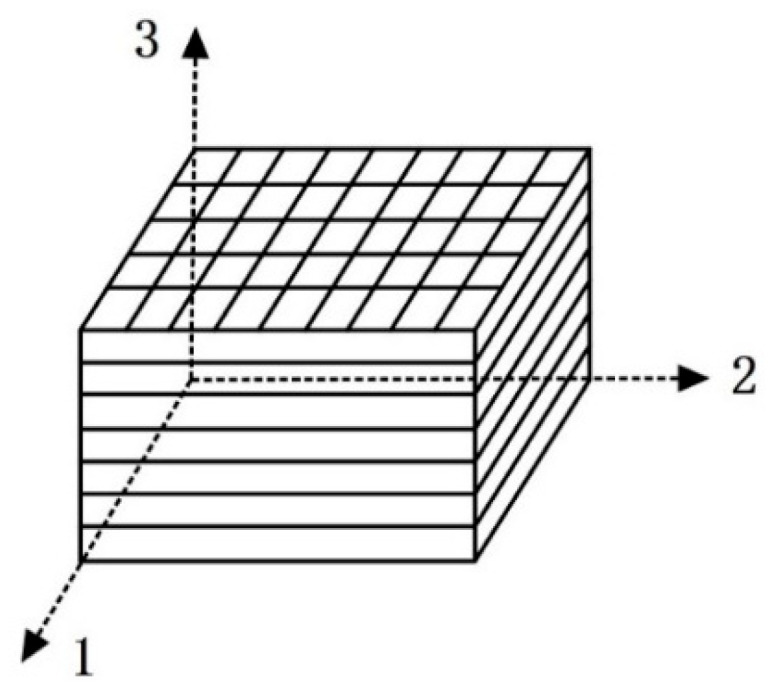
Coordinate system diagram of fiber reinforced composite.

The self-healing object of this study was a glass-fiber-reinforced polymer composite. This material is composed of 0°/90° biaxial glass fiber stitched fabric and epoxy resin, which is prepared via a vacuum-assisted resin transfer molding process and has orthogonal anisotropic mechanical properties [26]. In the Cartesian coordinate system describing the principal axis direction of the fiber-reinforced composite, the three mechanical principal axes are glass fiber longitudinal 1 (x-axis direction), glass fiber transverse 2 (y-axis direction), and the lamination direction, or normal 3 (thickness, z-axis direction), as shown in Figure 1.

**Figure 2:**
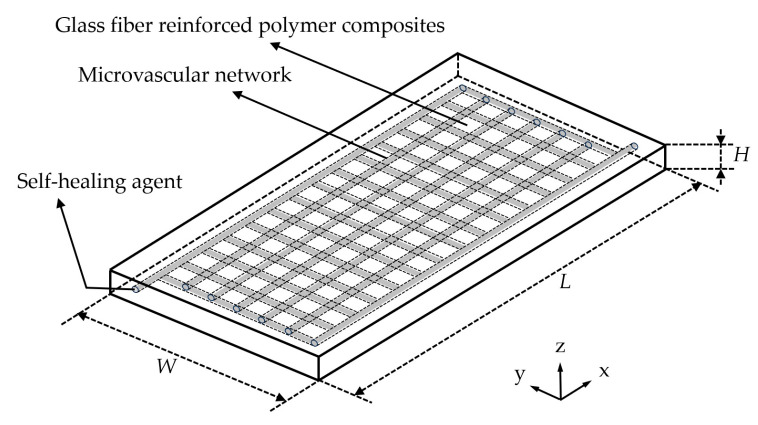
Self-healing structural model for orthotropic anisotropic composites.

The self-healing structural model consists of three parts: the glass-fiber-reinforced polymer composite, the monolayer orthogonal wall-less microvascular network implanted in the composite material after optimization, and the self-healing agent within the microvascular network, as shown in Figure 2. *L*, *W* and *H* are the length, width and height of the self-healing structural, respectively.

### 2.2. Microvascular Network Carrier Structure Optimization

**Figure 3:**
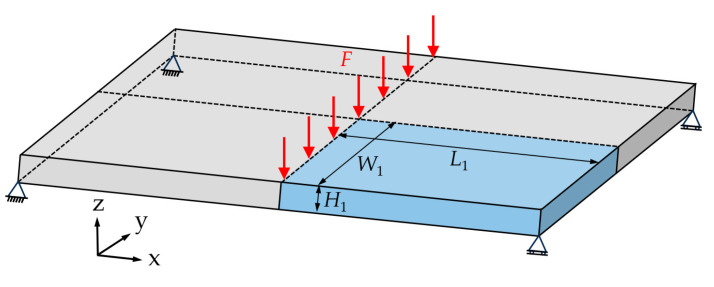
Computational example of the self-healing structural optimization of orthotropic anisotropic composites.

Figure 3 shows an example of self-healing structural optimization. In the structure, the horizontal and vertical microvascular are laid from the middle to both sides at equal intervals. In order to reduce the computational burden of topology optimization, considering the symmetry of the structure of the optimization example, the overall optimization is achieved by optimizing the light blue part (1/4 structure) in Figure 3.

The dimensions of the 1/4 structural design domain are *L*_1_ = 40 mm, *W*_1_ = 10 mm, and *H*_1_ = 2 mm. The 1/4 structural design domain is discretized using a uniform mesh of 200 × 50 × 10 eight-node quadrilateral elements. A uniformly distributed load of *F* = 50 N is applied to 50 adjacent finite element mesh vertices near the upper-left corner of the 1/4 structural design domain, close to the axis of symmetry. The loaded area forms a line segment containing 51 finite element mesh vertices. Hinge constraints were applied to the left side (constraining the x-axis directions), back (constraining the y-axis directions), and the bottom-right corner (constraining the x, y and z-axis directions) of the 1/4 structure.

The elastic constants of orthotropic anisotropic composites are shown in Table 1 [27].

In order to ensure the overall mechanical properties and self-healing performance of the self-healing structure, in this study, the longitudinal and transverse distribution spacing parameters *α* and *β*, respectively, and radius r of the microvascular were used as the optimization parameters, and three optimization objectives were set: (1) the compliance of the self-healing structure *f*_1_; (2) the water head loss *f*_2_; and (3) the volume percentage of microvascular network *f*_3_.

#### 2.2.1. Objective Function I: Self-Healing Structural Compliance

Compliance characterizes the mechanical properties of the structure. The smaller the compliance value of the self-healing structure, the greater the deformation resistance to external forces, and the better the stability of the overall structure. Therefore, in this study, the compliance of the self-healing structure was taken as the objective function I, and eight-node linear interpolation was used for the finite element analysis of the topology. Then, we solve for the objective function I by taking the derivative of the multivariate function and the chain rule.

First of all, based on the MMC method [28,29], the topological description of the self-healing structure is established as follows:

(1) $$\left\{\begin{array}{cc} \varphi_{i}(x,y,z)>0, & if\;(x,y,z)\in \Omega_{i},\\ \varphi_{i}(x,y,z)=0, & if\;(x,y,z)\in \partial \Omega_{i},\\ \varphi_{i}(x,y,z)<0, & if\;(x,y,z)\in D\backslash \Omega_{i}. \end{array}\right.$$

In Formula (1), *D* denotes the design domain of the specified, $\Omega_{i}$ denotes the region occupied by the *i*-th microvascular, and $\partial \Omega_{i}$ denotes the edge of the structure.

In order to realize the optimization of the monolayer orthogonal wall-less microvascular network, this study constructs the topology description function (TDF) of 3D components similar to prisms based on Eulerin description. In the 1/4 structural design domain, the TDF is used to express the microvascular explicitly, so that the microvascular can be bonded to form an interconnected network structure. Among them, the expression for the microvascular parallel to the x-axis is as follows:

(2) $$\varphi (x,y,z)=1-{\left(\frac{x’}{L/2}\right)}^{p}-{\left(\left.\frac{y’}{r}\right)\right.}^{p}-{\left(\frac{z’}{r}\right)}^{p}$$

The expression for the microvascular parallel to the y-axis is as follows:

(3) $$\varphi (x,y,z)=1-{\left(\frac{x’}{r}\right)}^{p}-{\left(\frac{y’}{W/2}\right)}^{p}-{\left(\frac{z’}{r}\right)}^{p}$$

(4) $$n_{1}=number\left(0:\alpha \cdot d_{e}:L-5d_{e}\right)$$

(5) $$n_{2}=number\left(W:-\beta \cdot d_{e}:5d_{e}\right)$$

(6) $$n=n_{1}+n_{2}$$

(7) $$\left\{\begin{array}{c} x^{\prime }\\ y^{\prime }\\ z^{\prime } \end{array}\right\}=\left[\begin{array}{ccc} c_{b}^{i}\cdot c_{t}^{i} & -c_{b}^{i}\cdot s_{t}^{i} & s_{b}^{i}\\ s_{a}^{i}\cdot s_{b}^{i}\cdot c_{t}^{i}+c_{a}^{i}\cdot s_{t}^{i} & -s_{a}^{i}\cdot s_{b}^{i}\cdot s_{t}^{i}+c_{a}^{i}\cdot c_{t}^{i} & -s_{a}^{i}\cdot c_{b}^{i}\\ -c_{a}^{i}\cdot s_{b}^{i}\cdot c_{t}^{i}+s_{a}^{i}\cdot s_{t}^{i} & c_{a}^{i}\cdot s_{b}^{i}\cdot s_{t}^{i}+s_{a}^{i}\cdot c_{t}^{i} & c_{a}^{i}\cdot c_{b}^{i} \end{array}\right]\left\{\begin{array}{c} x-x_{0}^{i}\\ y-y_{0}^{i}\\ z-z_{0}^{i} \end{array}\right\}$$

In Formulas (2)–(7), *r* denotes the radius of the microvascular; *p* = 6; *d_e_* denotes a finite element mesh size; and *α* and *β* denote the finite element cell spacing between the axes of each microvascular in the x and y axis directions, respectively, which has an important influence on the density of microvascular network layout. *L*/2 and *W*/2 denote the semi-axis length of the pipe in the x and y directions, respectively. *n*_1_ denotes the number of microvascular channels parallel to the y-axis; *n*_2_ denotes the number of microvascular channels parallel to the x-axis; and *number* indicates the number of arrays. Formulas (4) and (5) take 5 times the cell size *de* as the end point, the purpose of which is to prevent the feature size of the microvascular structure from exceeding the design domain. $s_{a},\;s_{b}$, and $s_{t}$ denote the sinusoidal angles of the component in the x, y, and z-axis, respectively. $c_{a}=\sqrt{1-{\left(s_{a}\right)}^{2}}$, $c_{b}=\sqrt{1-{\left(s_{b}\right)}^{2}}$, and $c_{b}=\sqrt{1-{\left(s_{b}\right)}^{2}}$.

In the design domain of the self-healing structure, the topology description of the microvascular network is expressed as follows:

(8) $$\varphi^{S_{1}}\left(x,y,z\right)=\max\left(\varphi_{1}\left(x,y,z\right),\ldots ,\varphi_{i}\left(x,y,z\right),\ldots ,\varphi_{n}\left(x,y,z\right)\right)$$

In Formula (8), $\varphi_{i}\left(x,y,z\right)$ denotes the topological description function of the *i*-th microvascular assembly.

For the 1/4 structural design domain, the topological description function of the self-healing macro structure is

(9) $$\varphi^{S_{2}}(x,y,z)=1-{\left(\frac{x}{L/2}\right)}^{p}-{\left(\frac{y}{W/2}\right)}^{p}-{\left(\frac{z}{H/2}\right)}^{p}$$

In Formula (9), *L*, *W*, and *H* denote the length, width and height of the self-healing macro structure, respectively.

The self-healing structure topology of orthotropic anisotropic composites $\varphi^{d}\left(x,y,z\right)$ is described as follows:

(10) $$\varphi^{d}\left(x,y,z\right)=-\max\left(\varphi^{S_{1}}\left(x,y,z\right),-\varphi^{S_{2}}\left(x,y,z\right)\right)$$

In Formula (10), $\varphi^{S_{1}}\left(x,y,z\right)$ describes the microvascular network topology of self-healing materials; $\varphi^{S_{2}}\left(x,y,z\right)$ describes the macroscopic topology of the self-healing material; and $\varphi^{d}\left(x,y,z\right)$ describes the overall topology of the self-healing material. The design variable of the overall structure is $D=\left(x,y,z,r,\alpha ,\beta \right)$.

Secondly, this study takes orthotropic anisotropic composites as self-healing objects. Under three-dimensional strain conditions, orthotropic anisotropic composites have the following stress–strain relationship [26]:

(11) $$\left(\begin{array}{l} \varepsilon_{11}\\ \varepsilon_{22}\\ \varepsilon_{33}\\ \gamma_{23}\\ \gamma_{31}\\ \gamma_{12} \end{array}\right)=\left(\begin{array}{cccccc} S_{11} & S_{12} & S_{13} & 0 & 0 & 0\\ S_{21} & S_{22} & S_{23} & 0 & 0 & 0\\ S_{31} & S_{32} & S_{33} & 0 & 0 & 0\\ 0 & 0 & 0 & S_{44} & 0 & 0\\ 0 & 0 & 0 & 0 & S_{55} & 0\\ 0 & 0 & 0 & 0 & 0 & S_{66} \end{array}\right)\left(\begin{array}{l} \sigma_{11}\\ \sigma_{22}\\ \sigma_{33}\\ \sigma_{23}\\ \sigma_{31}\\ \sigma_{12} \end{array}\right)$$

In Formula (11), *S_ij_* is the compliance matrix coefficient and *S* is the compliance matrix, whose component is determined by the elastic modulus, Poisson’s ratio, and shear modulus of the composite material along the three main axes and is the inverse matrix of the stiffness matrix, which is used to calculate the compliance value of the orthotropic material. The relationship between the compliance matrix *S* of orthotropic anisotropic composites and the 9 elastic constants [30] is shown in Equation (12):

(12) $$S=\left(\begin{array}{cccccc} \frac{1}{E_{11}} & -\frac{u_{21}}{E_{22}} & -\frac{u_{31}}{E_{33}} & 0 & 0 & 0\\ -\frac{u_{12}}{E_{11}} & \frac{1}{E_{22}} & -\frac{u_{32}}{E_{33}} & 0 & 0 & 0\\ -\frac{u_{13}}{E_{11}} & -\frac{u_{23}}{E_{22}} & \frac{1}{E_{33}} & 0 & 0 & 0\\ 0 & 0 & 0 & \frac{1}{G_{23}} & 0 & 0\\ 0 & 0 & 0 & 0 & \frac{1}{G_{13}} & 0\\ 0 & 0 & 0 & 0 & 0 & \frac{1}{G_{12}} \end{array}\right)$$

Therefore, the formula for calculating the compliance of the self-healing structure of the built-in monolayer orthogonal wall-less microvascular network carrier is as follows [31]:

(13) $$f_{1}=C=\int_{D}H(\varphi^{d}(x,y,z;D))f\cdot udV+\int_{\Gamma_{t}}t\cdot udS$$

In Formula (13), *C* denotes compliance. *f* and *t* denote the volume force on the structure and the surface force on the Neumann boundary $\Gamma_{t}$, respectively. *u* denotes the displacement field of the self-healing structure of the built-in microvascular network carrier. H(x) stands for the Heaviside function, which is expressed in the following form [32]:

(14) $$H_{\varepsilon }(x)=\left\{\begin{array}{c} 1,\\ \frac{3(1-\upsilon )}{4}(\frac{x}{\varepsilon }-\frac{x^{3}}{3\varepsilon^{3}})+\frac{\left(1+\upsilon \right)}{2},\\ \upsilon , \end{array}\right.\begin{array}{cc} & if\begin{array}{c} x>\varepsilon \end{array}\\ & if\begin{array}{c} -\varepsilon \leq x\leq \varepsilon \end{array}\\ & otherwise \end{array}$$

In Formula (14), *ε* represents the parameter of regularization, which is to control the width of regularization. υ represents a very small positive number that prevents the overall stiffness matrix from being singular.

#### 2.2.2. Objective Function II: Water Head Loss

The water head loss of the microvascular network characterizes the flow efficiency of the healing agent in the pipe. Different from the calculation of compliance of objective function I, the calculation of water head loss in this study takes the overall pipe network as the object, and its calculation formula is as follows:

(15) $$f_{2}=\lambda \sum_{k}^{n_{p}}\frac{l_{k}}{d}\frac{v_{k}^{2}}{2g}$$

In Formula (15), λ denotes the hydraulic resistance coefficient, *n_p_* denotes the number of microvascular segment, *l_k_* denotes the length of the *k*-th microvascular segment, *v_k_* denotes the flow rate of liquid in the *k*-th microvascular segment, *d* denotes the diameter of the microvascular segment, and *g* denotes the gravitational acceleration, which is 9.8 m/s^2^.

#### 2.2.3. Objective Function III: Volume Percentage of Microvascular Network

The high volume percentage of a microvascular network can ensure the completeness and efficiency of the material repair, and can also affect the mechanical properties of the material. Therefore, it is necessary to reasonably control the volume percentage of the microvascular network. The formula for calculating the volume percentage of microvascular network is as follows:

(16) $$f_{3}=\frac{\sum_{k}^{n_{p}}l_{k}\cdot \pi r^{2}}{V}$$

In Formula (16), *l_k_* denotes the length of the *k*-th microvascular segment, *n_p_* denotes the number of microvascular segment, *r* denotes the radius of the microvascular, and *V* denotes the volume of the material.

### 2.3. Non-Dominated Sorting Genetic Algorithm II

In this study, the self-healing structural compliance, water head loss, and volume percentage of microvascular network were taken as the objective functions, and the non-dominated sorting genetic algorithm II (NSGA-II) [33,34] was used to optimize the distribution spacing parameters α and β and radius r of the transverse and vertical channels of the microvascular network. Due to the inconsistency of optimization directions among the multiple optimization objectives considered in this study, the concept of Pareto non-inferior solution [35] is introduced to comprehensively measure the quality of the optimized solution. Pareto non-inferior solutions are used to eliminate the disadvantageous solutions in all individuals. Therefore, the optimization algorithm based on Pareto is a set of multiple non-inferior solutions. The initial parameter settings of the NSGA-II optimization algorithm are shown in Table 2.

The specific steps of the algorithm are as follows:

Step 1: An initial population *P* with size |*P*| is randomly generated.

Step 2: The objective functions *f*_1_, *f*_2_, and *f*_3_ of all individuals in population *P* are calculated and non-dominated ordering is performed.

Step 3: Individuals are selected via the tournament method to generate the parent population *E*_t_ (t = 0) with size |*E*_t_|.

Step 4: The parent population *E*_t_ is crossed and mutated to generate the offspring population *Q*_t_ with size 3|*E*_t_|.

Step 5: The parent population and offspring population are combined to obtain the combined population *F*_t_ = *Q*_t_ ∪ *E*_t_.

Step 6: All the individuals in the combined population *F*_t_ are quickly non-dominated sorted to obtain the non-dominated sorted levels *K*_1_, *K*_2_, …, *K*_n_. The crowding distance of all individuals in each *K_i_* level is calculated and sorted from large to small.

Step 7: The elite strategy [36] is adopted to select the size |*E*_t_| from the merged population *F*_t_ as the new population *E*_t+1_.

Step 8: It is determined whether the iteration meets the termination condition—that is, the results are consistent for 5 successive generations, *K*_1_^t^ = *K*_1_^t−1^ = … = *K*_1_^t−4^, or the maximum number of iterations is reached, t > 300. If yes, all individuals corresponding to the Pareto frontier solution set *K*_1_ are output. Otherwise, the number of iterations increases by 1, that is, t = t + 1, and the algorithm jumps to step 4 and continues to run.

**Figure 4:**
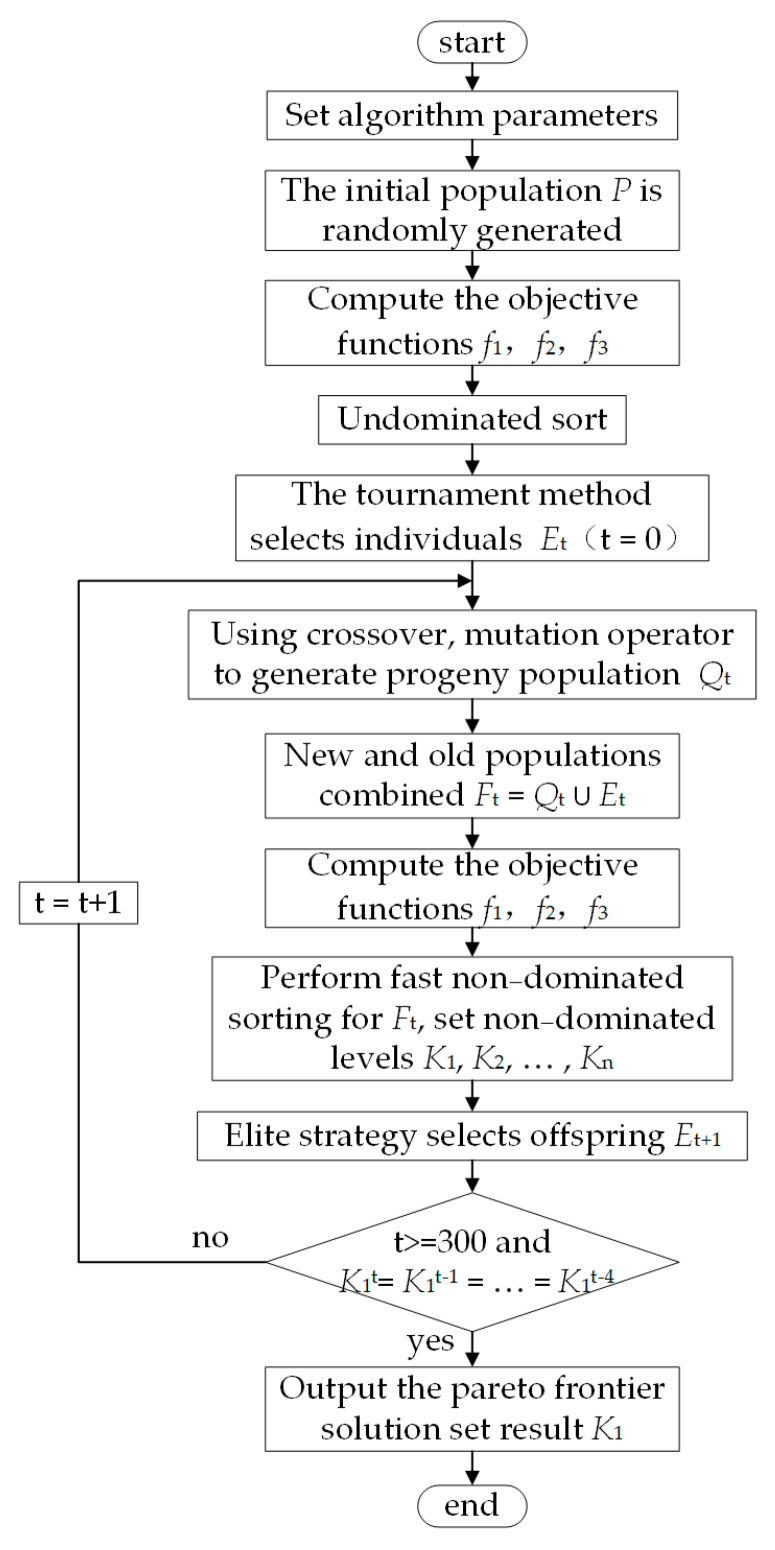
Flow chart of NSGA-II.

The algorithm flow is shown in Figure 4.

### 2.4. Microvascular Network Optimization Results

**Figure 5:**
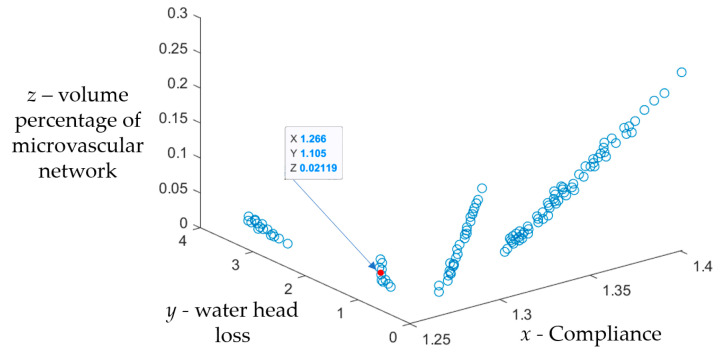
Optimization results of the NSGA-II algorithm.

Figure 5 shows the optimization results of the NSGA-II algorithm. The first layer of non-inferior solution set *K*_1_ contains 119 solutions. According to the optimization results,

(1) The embedding of the optimized microvascular network has little effect on the mechanical properties of the material. Compared with the material structure without a microvascular network, the compliance of the self-healing structure of the built-in monolayer wall-less microvascular network decreased to a certain extent, and the decrease ranged from 0.021% to 10.81%.
(2) When the microvascular spacing parameters *α* and *β* remain unchanged, with the increase in the radius *r* of the microvascular structure, the overall compliance of the self-healing structure increases, the water head loss of the microvascular network decreases, and the volume percentage of the microvascular network increases.
(3) When the radius *r* of the microvascular is unchanged, the overall compliance of the self-healing structure decreases with the increase in the microvascular spacing parameters *α* and *β*, and the water head loss and the volume percentage of the microvascular network also decrease.

## 3. Self-Healing Structure Preparation and Performance Study

**Figure 6:**
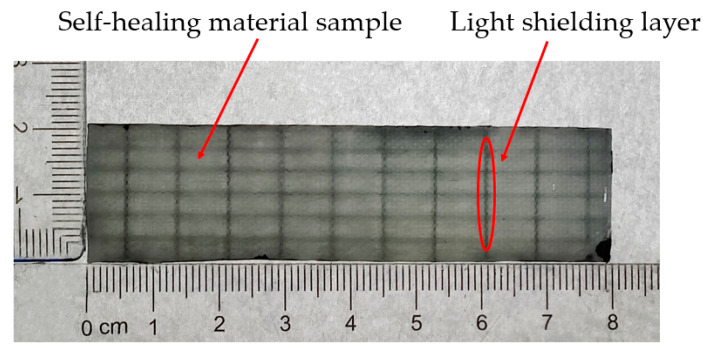
Self-healing material samples.

In order to verify the effectiveness of the method for the topology optimization of self-healing structures for orthotropic anisotropic composites, this study firstly selected the suitable Pareto non-inferior solution (the solution indicated by the arrow in Figure 6; the parameters are shown in Table 3) to develop the microvascular network carrier based on the topology optimization results combined with the preparation operability. Secondly, the self-healing material specimen was prepared by using the vacuum-assisted resin transfer molding (VARTM) process and pre-embedded wire removal method. Finally, mechanical tests and self-healing tests were carried out to verify the effectiveness of the optimization method and evaluate the self-healing effect.

### 3.1. Preparation of Monolayer Wall-Less Microvascular Network Self-Healing Materials

#### 3.1.1. Raw Materials and Reagents

The raw materials and reagents for preparing the self-healing material sample are shown in Table 4.

#### 3.1.2. Self-Healing Material Specimen Preparation Steps

According to the optimized parameters (Table 3), the standard size of the prepared sample was 80 × 20 × 2 mm, the microvascular network diameter was 0.4 mm, and the transverse and longitudinal intervals between the microvascular channels were *α* = 7.8 mm and *β* = 3.4 mm, respectively. The specific experimental steps were as follows:

First, the glass fiber cloth reinforcement material was laid and the microvascular network sacrifice line was embedded, and the sacrifice line was coated with release agent, which helped to maintain the integrity of the microvascular network structure during the removal process and the preparation of the epoxy resin mixed solution. A vacuum pump was used to pump the epoxy resin mixture into the glass-fiber-cloth-reinforced material between the layers and completely infiltrate it, waiting for curing at room temperature for 48 h. Secondly, the sacrifice line of the microvascular network was mechanically removed, and the material was further cut and processed into standard-size samples. Finally, the black epoxy resin coating was used as a light shielding agent, which was coated on the inner wall of the pipe and cured for 24 h to form a light shielding layer of the microvascular network. The light repair agent was injected into the microvascular network to produce self-healing materials.

A sample of the self-healing material containing the monolayer wall-less microvascular network is shown in Figure 6.

### 3.2. Mechanical Properties and Light Repair Properties of Self-Healing Materials

In this study, the three-point bending test was used to test the material properties, and the bending strength was used as the evaluation index of mechanical properties of self-healing materials. The standard and related parameters of the three-point bending test were as follows: the sample was placed in the middle position of the three-point bending test device, and the span was adjusted to 50 mm to ensure that the middle symmetric axis position of the sample was subjected to force. During the test, the displacement control mode was used, the waveform was selected as oblique waves, and the test speed was set at 0.5 mm/s. Two groups of comparisons were made in this experiment: (1) focusing on the influence of the built-in microvascular network carrier on the overall performance of the structure, the bending strength changes in the samples with and without the built-in microvascular network carrier were compared; (2) regarding the self-healing properties, the bending strength changes in the self-healing specimens with and without light repair were compared.

In this experiment, three groups of specimens were prepared, including group A without microvascular network carrier samples, group B with microvascular network carrier samples but without a repair agent, and group C with microvascular network carrier samples and a light repair agent. Each group contained five specimens. The mechanical properties of the self-healing materials were analyzed by comparing the specimens in group A and group B (the self-healing of light energy was not introduced in the experiment). The light repair properties of the self-healing materials were analyzed by comparing the samples in group B and group C.

#### 3.2.1. Mechanical Property Test

The mechanical properties of the self-healing materials were compared and analyzed by means of the three-point bending test of the specimens in group A and group B. The loading speed was 0.5 mm/min, and the bending strength change of the specimen during the loading process was tested. According to the load–displacement curve obtained during the three-point bending test, the specimen underwent the elastic process (no failure), yield process (partial damage), and failure (complete damage). We define the peak of the entire curve during this period as the maximum failure load, and the second peak of the curve thereafter as the second peak load.

The test results (as shown in Table 5) show that the average maximum failure load of the samples without microvascular network carrier is 1.3700 kN, while the average maximum failure load of the samples with microvascular network carrier is 1.2886 kN. The average failure load of the self-healing sample is 94.06% of that of the samples without microvascular network carriers. The results show that the addition of microvascular network carriers will not significantly worsen the mechanical properties of the material, and it also reflects the effectiveness of the optimization method of microvascular network carriers.

#### 3.2.2. Light Repair Property Test

**Figure 7:**
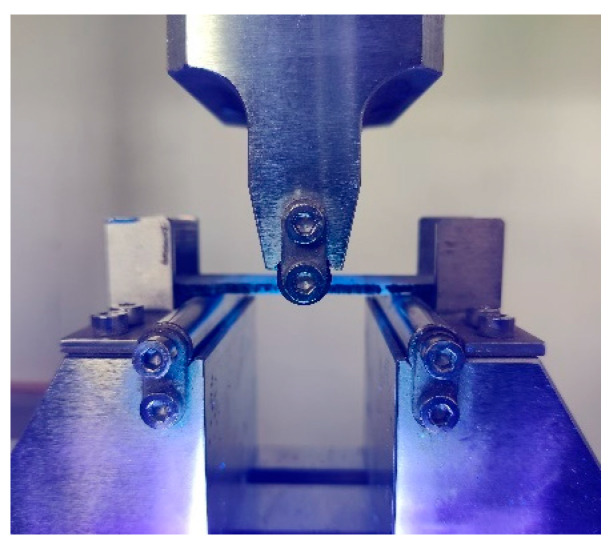
Three-point bending test for light energy repair.

The three-point bending test was carried out on group C samples, and compared with the test results of group B samples, the light repairing performance was analyzed. The loading speed was 0.5 mm/min. Before the experiment, the photosensitive resin DAZZLE LV305 was injected into the microvascular network of the sample as a light repair agent. Compared with other self-healing agents, the photosensitive resin used in this study had lower viscosity, shorter curing time, good fluidity in the microvascular network, and complete curing could be achieved via direct irradiation at a distance of 20 mm from the short-wave light source for 10 s. During the experiment, a UV curing light source (10 W, 365~410 nm) was used to stimulate the light repair (as shown in Figure 7).

**Figure 8:**
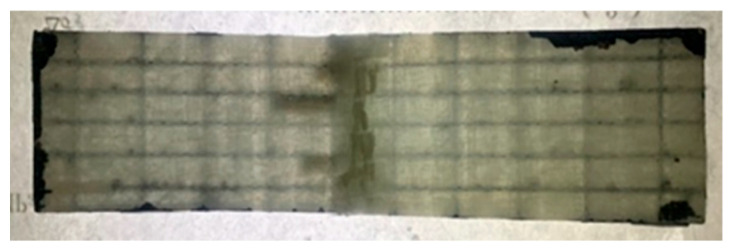
Physical picture of the specimen after light repair test.

The sample tested by means of the light-repaired three-point bending test is shown in Figure 8.

From the results of the three-point bending test in Table 5 and Figure 8, the recorded data of group C samples are generally higher than those for group B samples. The experimental results show that:

(1) Figure 8 shows the sample front drawing after the test, from which it can be seen that there is an obvious curing phenomenon in the middle of the sample. This indicated that the microvascular network of the specimen at the compression site ruptured, leading to the outflow of the light repair agent. Under the irradiation of excitation light, the light repair agent produces a photochemical reaction, which causes it to cure in a short time and repairs the damaged part of the material.
(2) The average maximum failure load of group B samples is 1.2886 kN, while that of group C samples is 1.3410 kN. Compared with the specimens without light repair, the average maximum failure load of the specimens with light repair was increased by 4.1%. The reason for this is that with the application of load, small cracks continue to appear in the sample, which induces the rupture of the microvascular network and the outflow of the repair agent. The repair agent cured under the light excitation, and the self-healing of the damaged crack was realized, and thus the peak load of the specimen was increased.
(3) After the sample loading passed the load peak, the second peak load of group B samples was 0.5358 kN when the sample continued to be loaded until the complete fracture of the material occurred. The second peak load of group C samples was 0.6888 kN, which reached 51.36% of the average maximum failure load, and was 28.56% higher than that of group B samples.
(4) Compared with group A samples, the average maximum load of group C samples is 97.88% of that of group A samples, while that of group B samples is 94.06% of that of group A samples. The experimental data show that microvascular network embedding affects the failure mechanism, and the introduction of light repair can eliminate the above effects. The data also showed the effectiveness of the optimization, and also reflected that the light repair agent had a repairing effect on the material during the test.
(5) In this experiment, the whole process of self-healing was completed during a three-point test, so the real-time nature of light repair was demonstrated.

According to the above experimental data, it can be proved that the damage light repair of the sample has good real-time performance, and it can quickly complete the damage self-healing of light energy.

## 4. Conclusions

Aiming at the self-healing research of orthotropic anisotropic composites, a self-healing structure based on monolayer wall-less microvascular network carriers for orthotropic anisotropic composites was proposed. The radius and spacing of the microvascular network were optimized based on NSGA-II. A wall-less microvascular network was prepared by means of the embedded wire removal method. Light repair technology is used to realize the self-healing of material damage. The three-point bending test successfully proves that the optimization of the microvascular network helps to improve the mechanical compatibility of the materials, and also verifies the effectiveness of the light repair, which can achieve the real-time and rapid repair of the materials.

(1) The study takes orthotropic anisotropic glass-fiber-reinforced polymer composites as the repair object and establishes the stress–strain relationship matrix of these orthotropic anisotropic composites. It introduces a topological descriptive function and conducts finite element analysis to solve the compliance of the structure. Taking the self-healing structural compliance, water head loss, and volume percentage of the microvascular network as the objectives, the NSGA-II algorithm was used to optimize the topology of the microvascular network, and the effectiveness of the optimization method was verified by experiments.
(2) Based on the vacuum-assisted resin transfer molding process and the embedded wire removal method, the self-healing material samples with built-in monolayer wall-less microvascular network carriers were prepared, and the mechanical properties of the materials were tested by means of the three-point bending test. The results showed that the average maximum failure load of the self-healing material was 94.06% of that of the sample without carriers. The experimental data reflect the effectiveness of the microvascular network carrier optimization method, and this optimization method will help to improve the mechanical compatibility of the materials.
(3) In this study, a single-component short-wave light-curing resin was used as a light repair agent to achieve the on-line and real-time self-healing of damage. Compared with other restorative agents, its light curing reaction is rapid and self-healing of material damage can be achieved quickly. The bending test results show that in the case of the introduction of real-time light energy repair, the average maximum failure load of the structure with light repair was increased by 4.1% compared to that without light repair. After passing through the peak load, the second peak load of the structure with light repair can reach 51.36% of the average maximum failure load, which is 28.56% higher than that of the structure without light repair.

In this study, we have conducted some work on the self-healing structure design of orthotropic composites, but we believe that further research is still needed. This is mainly reflected in the following aspects: the optimal design of self-healing structures can be extended to uncertain load conditions, and the influence of variable-angle fibers on self-healing structures can be considered.

## Figures and Tables

**Table 1:** Elastic constants of orthotropic anisotropic composites.

Modulus of Elasticity/GPa	Poisson’s Ratio	Shear Modulus/GPa
*E*_1_ = 16.3	*μ*_12_ = 0.058	*G*_12_ = 1.49
*E*_2_ = 16.6	*μ*_23_ = 0.282	*G*_23_ = 1.17
*E*_3_ = 3.0	*μ*_13_ = 0.262	*G*_13_ = 1.20

**Table 2:** Parameters of NSGA-II.

Parameters	Value
Initial population size |*P*|	100
Cross probability *P_cross_*	0.8
Variation probability *P_mutate_*	0.2
Parent and offspring population size |*E*_t_|	50
Model length *L*_1_/mm	40
Model width *W*_1_/mm	10
Model thickness *H*_1_/mm	2
Input port flow *V*_1_/mm^3^/s	100
Microvascular radius *r*/mm	{0.15, 0.2, 0.25, 0.3, 0.35, 0.4, 0.45}
Microvascular network spacing parameters *α* and *β*/mm	{15, 17, 19, 21, 23, 25, 27, 29, 31, 33, 35, 37, 39, 41, 43, 45}

**Table 3:** Microvascular network optimization scheme.

*α*	*β*	*r*/mm	Compliance	Water Head Loss/m	Volume Percentage of Microvascular Network/%
39	17	0.2	1.2662	1.1048	2.1190

**Table 4:** Raw materials and reagents.

Name	Model Number	Production and Sales Factory
Epoxy resin	Bisphenol A E51	Hangzhou Wuhuigang Adhesive Co., Ltd., Hangzhou, China
Modified amine epoxy curing agent	JH593	Hangzhou Wuhuigang Adhesive Co., Ltd., Hangzhou, China
0°/90° biaxial glass fiber stitched fabric	LT650-1340	Changzhou Hualike new Material Co., Ltd., Changzhou, China
Light repair agent	DAZZLE-LV305 150 ± 50 mPa·s (25 °C)	Shenzhen Daye laser forming technology Co., Ltd., Shenzhen, China
Light screening agent	two-component epoxy paint	Shanghai Jinsi Di metal paint factory, Shanghai, China

**Table 5:** Results from three-point bend flexural testing.

Specimen Group	Maximum Failure Load Range (kN)	Average Maximum Failure Load (kN)	Second Peak Load Range (kN)	Second Peak Load Mean (kN)	Percentage of Maximum Failure Load Achieved After Fracture
A	1.2450~1.4840	1.3700	—	—	—
B	1.1310~1.4700	1.2886	0.4190~0.6020	0.5358	41.58%
C	1.1810~1.5380	1.3410	0.6450~0.7170	0.6888	51.36%

## Data Availability

The original contributions presented in this study are included in the article. Further inquiries can be directed to the corresponding author.
